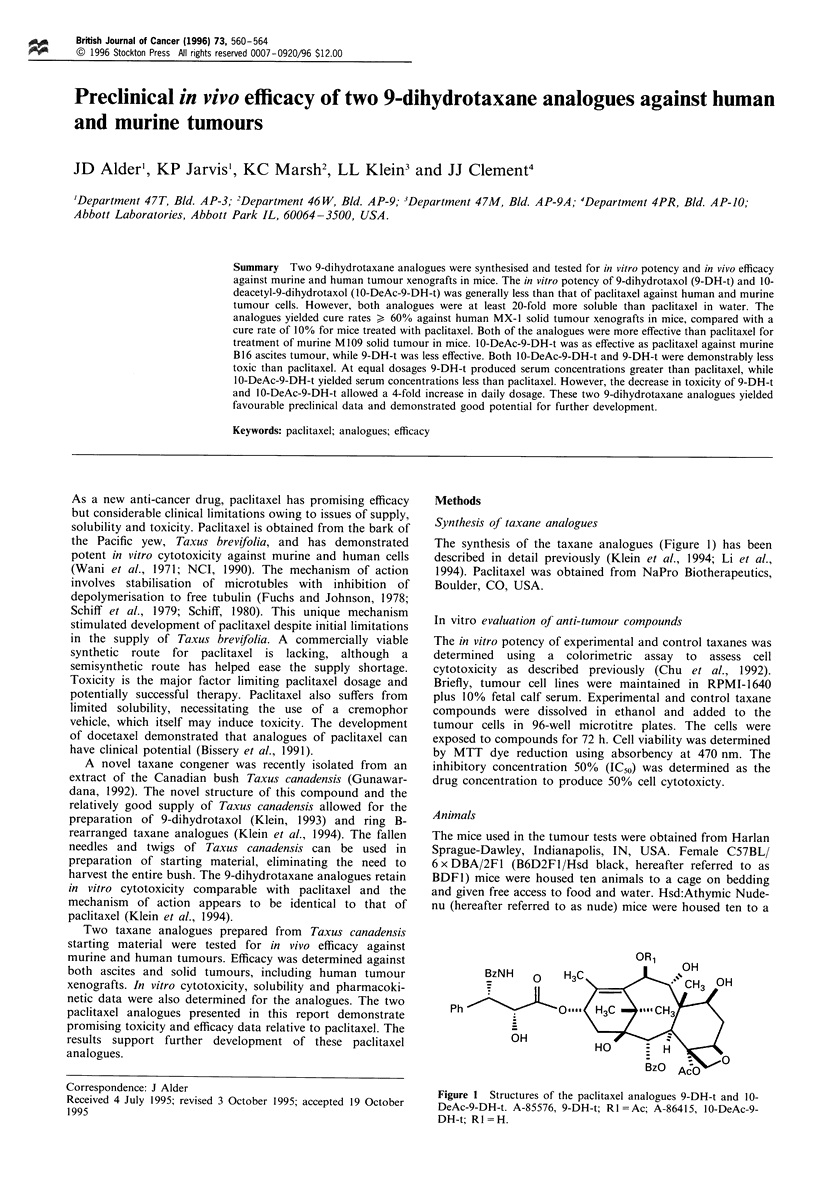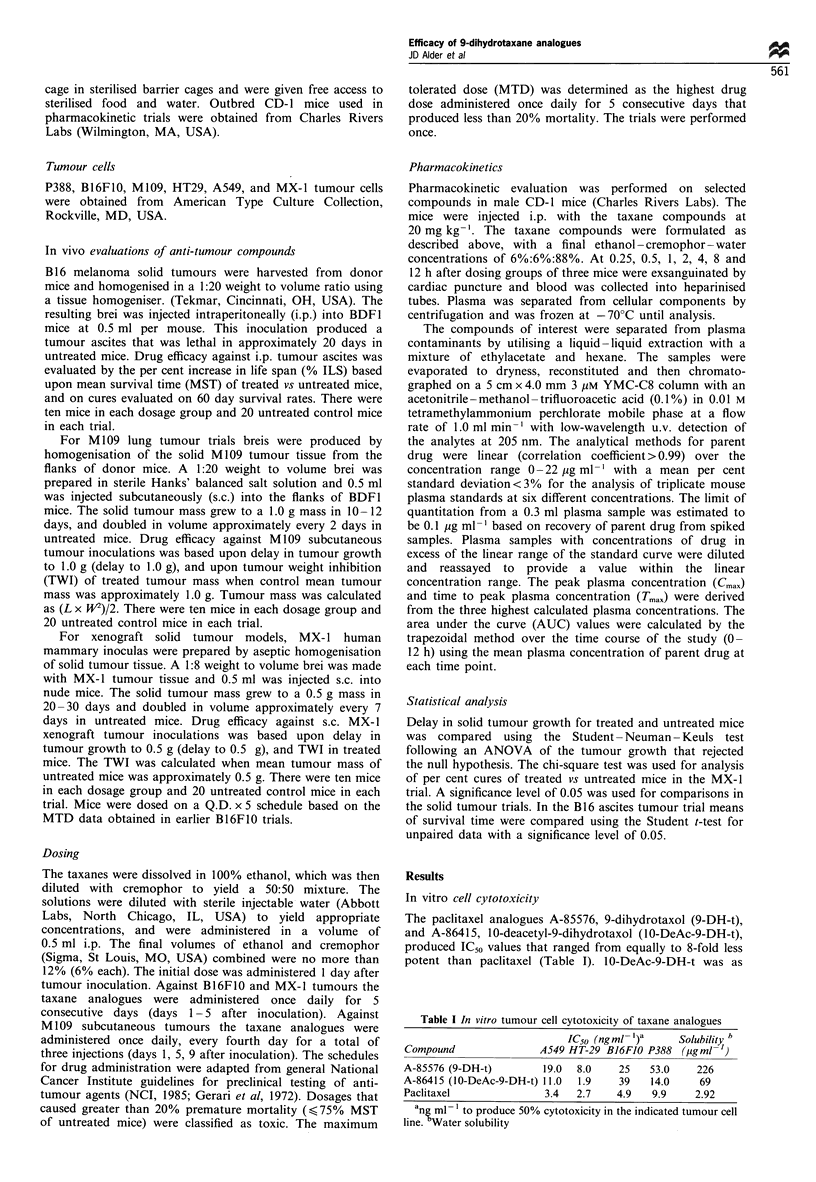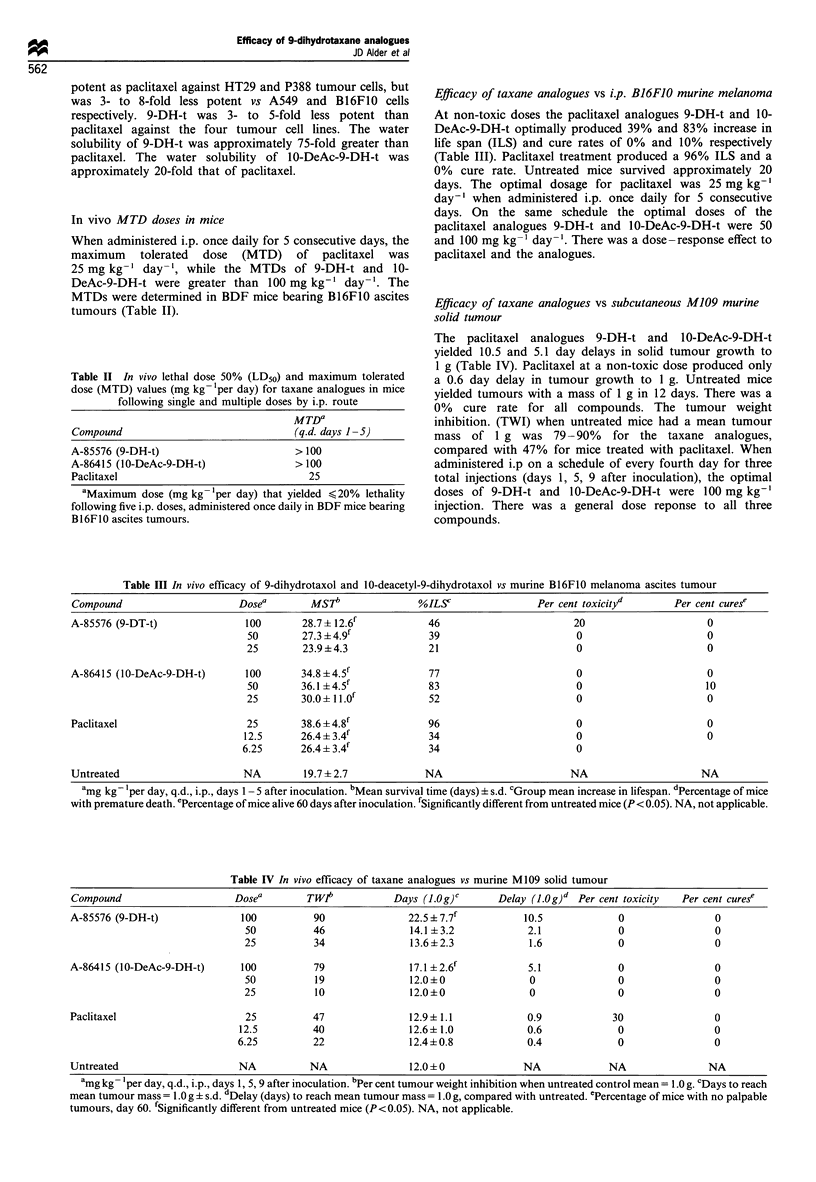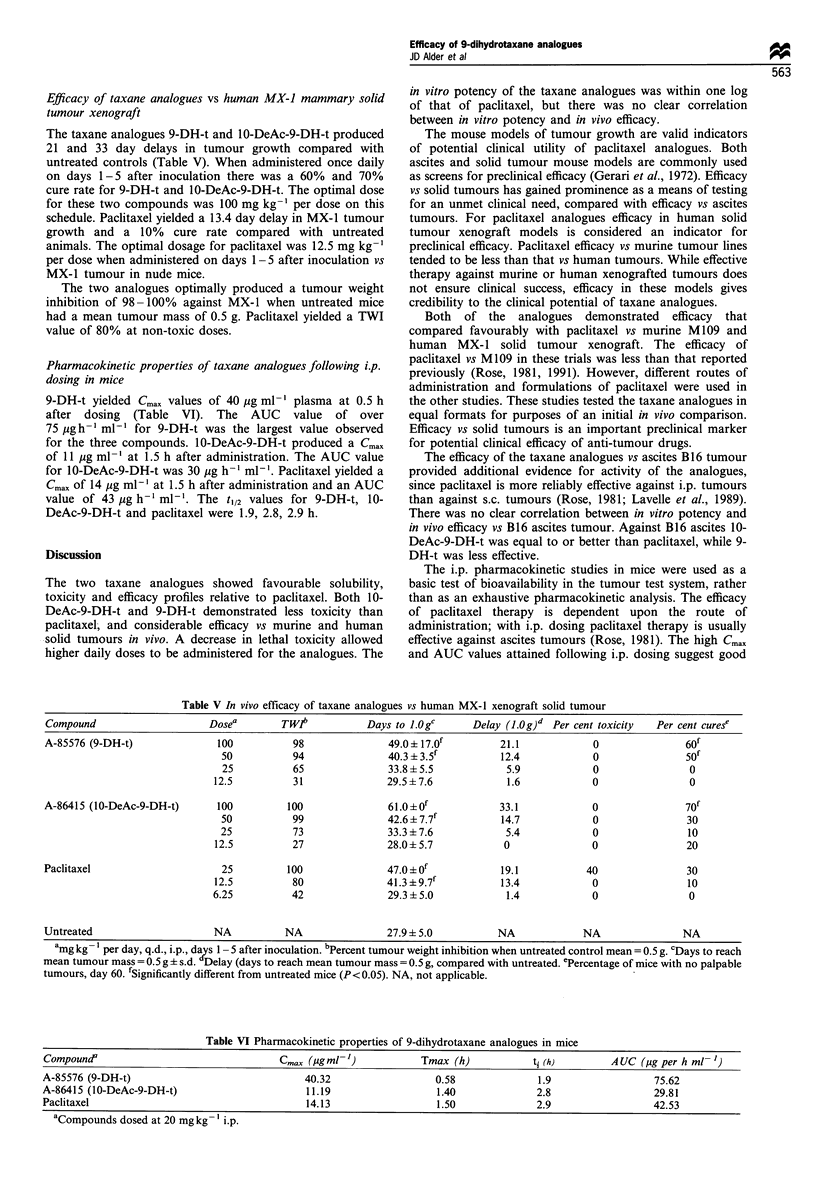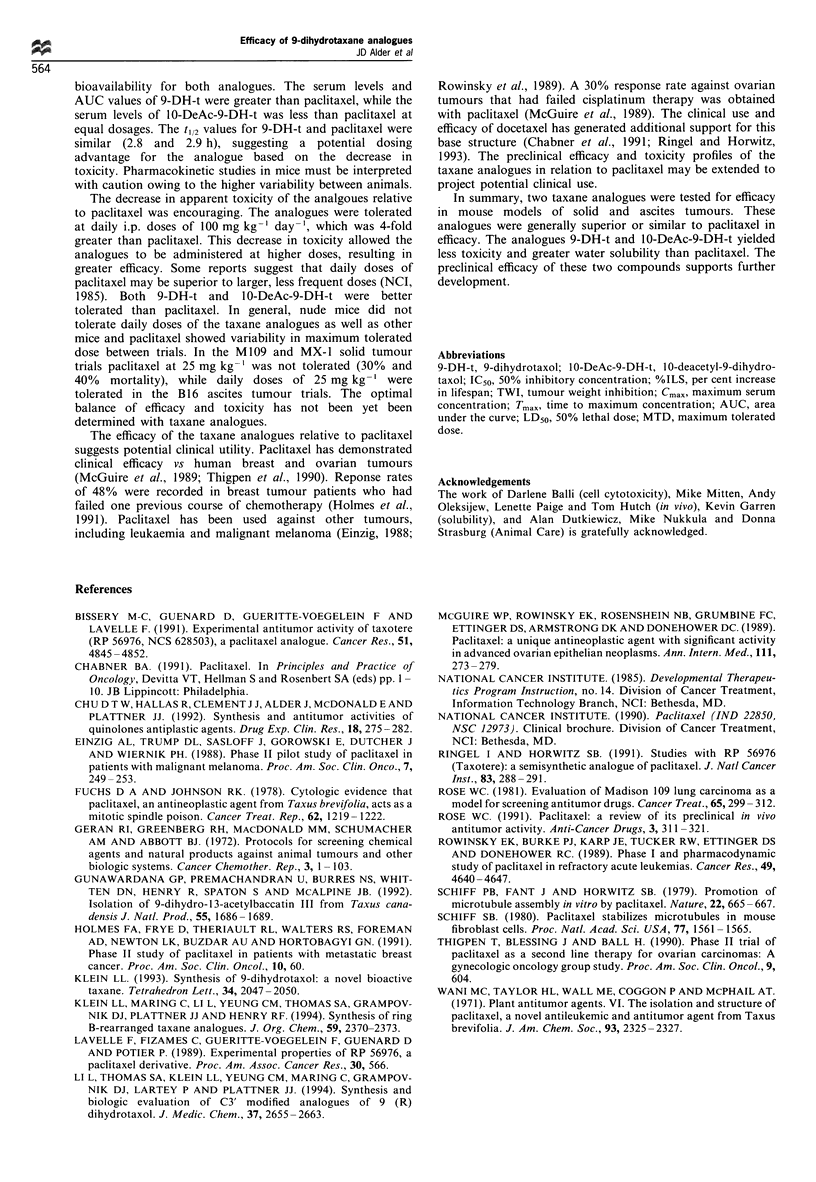# Preclinical in vivo efficacy of two 9-dihydrotaxane analogues against human and murine tumours.

**DOI:** 10.1038/bjc.1996.98

**Published:** 1996-03

**Authors:** J. D. Alder, K. P. Jarvis, K. C. Marsh, L. L. Klein, J. J. Clement

**Affiliations:** Abbott Laboratories, Abbott Park IL, 60064-3500, USA.

## Abstract

Two 9-dihydrotaxane analogues were synthesised and tested for in vitro potency and in vivo efficacy against murine and human tumour xenografts in mice. The in vitro potency of 9-dihydrotaxol (9-DH-t) and 10-deacetyl-9-dihydrotaxol (10-DeAc-9-DH-t) was generally less than that of paclitaxel against human and murine tumour cells. However, both analogues were at least 20-fold more soluble than paclitaxel in water. The analogues yielded cure rates > or = 60% against human MX-1 solid tumour xenografts in mice, compared with a cure rate of 10% for mice treated with paclitaxel. Both of the analogues were more effective than paclitaxel for treatment of murine M109 solid tumour in mice. 10-DeAc-9-DH-t was as effective as paclitaxel against murine B16 ascites tumour, while 9-DH-t was less effective. Both 10-DeAc-9-DH-t and 9-DH-t were demonstrably less toxic than paclitaxel. At equal dosages 9-DH-t produced serum concentrations greater than paclitaxel, while 10-DeAc-9-DH-t yielded serum concentrations less than paclitaxel. However, the decrease in toxicity of 9-DH-t and 10-DeAc-9-DH-t allowed a 4-fold increase in daily dosage. These two 9-dihydrotaxane analogues yielded favourable preclinical data and demonstrated good potential for further development.


British Journal of Cancer (1996) 73, 560-564
© 1996 Stockton Press All rights reserved 0007-0920/96 $12.00

### Preclinical *in vivo* efficacy of two 9-dihydrotaxane analogues against human and murine tumours

JD Alder[1], KP Jarvis[1], KC Marsh[2], LL Klein[3] and JJ Clement[4]

[1]*Department 47T, Bld. AP-3;* [2]*Department 46W, Bld. AP-9;* [3]*Department 47M, Bld. AP-9A;* [4]*Department 4PR, Bld. AP-10; Abbott Laboratories, Abbott Park IL, 60064-3500, USA.*

**Summary** Two 9-dihydrotaxane analogues were synthesised and tested for *in vitro* potency and *in vivo* efficacy against murine and human tumour xenografts in mice. The *in vitro* potency of 9-dihydrotaxol (9-DH-t) and 10-deacetyl-9-dihydrotaxol (10-DeAc-9-DH-t) was generally less than that of paclitaxel against human and murine tumour cells. However, both analogues were at least 20-fold more soluble than paclitaxel in water. The analogues yielded cure rates ⩾ 60% against human MX-1 solid tumour xenografts in mice, compared with a cure rate of 10% for mice treated with paclitaxel. Both of the analogues were more effective than paclitaxel for treatment of murine M109 solid tumour in mice. 10-DeAc-9-DH-t was as effective as paclitaxel against murine B16 ascites tumour, while 9-DH-t was less effective. Both 10-DeAc-9-DH-t and 9-DH-t were demonstrably less toxic than paclitaxel. At equal dosages 9-DH-t produced serum concentrations greater than paclitaxel, while 10-DeAc-9-DH-t yielded serum concentrations less than paclitaxel. However, the decrease in toxicity of 9-DH-t and 10-DeAc-9-DH-t allowed a 4-fold increase in daily dosage. These two 9-dihydrotaxane analogues yielded favourable preclinical data and demonstrated good potential for further development.

**Keywords:** paclitaxel; analogues; efficacy

As a new anti-cancer drug, paclitaxel has promising efficacy but considerable clinical limitations owing to issues of supply, solubility and toxicity. Paclitaxel is obtained from the bark of the Pacific yew, *Taxus brevifolia*, and has demonstrated potent *in vitro* cytotoxicity against murine and human cells (Wani *et al.*, 1971; NCI, 1990). The mechanism of action involves stabilisation of microtubles with inhibition of depolymerisation to free tubulin (Fuchs and Johnson, 1978; Schiff *et al.*, 1979; Schiff, 1980). This unique mechanism stimulated development of paclitaxel despite initial limitations in the supply of *Taxus brevifolia.* A commercially viable synthetic route for paclitaxel is lacking, although a semisynthetic route has helped ease the supply shortage. Toxicity is the major factor limiting paclitaxel dosage and potentially successful therapy. Paclitaxel also suffers from limited solubility, necessitating the use of a cremophor vehicle, which itself may induce toxicity. The development of docetaxel demonstrated that analogues of paclitaxel can have clinical potential (Bissery *et al.*, 1991).

A novel taxane congener was recently isolated from an extract of the Canadian bush *Taxus canadensis* (Gunawardana, 1992). The novel structure of this compound and the relatively good supply of *Taxus canadensis* allowed for the preparation of 9-dihydrotaxol (Klein, 1993) and ring B-rearranged taxane analogues (Klein *et al.*, 1994). The fallen needles and twigs of *Taxus canadensis* can be used in preparation of starting material, eliminating the need to harvest the entire bush. The 9-dihydrotaxane analogues retain *in vitro* cytotoxicity comparable with paclitaxel and the mechanism of action appears to be identical to that of paclitaxel (Klein *et al.*, 1994).

Two taxane analogues prepared from *Taxus canadensis* starting material were tested for *in vivo* efficacy against murine and human tumours. Efficacy was determined against both ascites and solid tumours, including human tumour xenografts. *In vitro* cytotoxicity, solubility and pharmacokinetic data were also determined for the analogues. The two paclitaxel analogues presented in this report demonstrate promising toxicity and efficacy data relative to paclitaxel. The results support further development of these paclitaxel analogues.

Correspondence: J Alder
Received 4 July 1995; revised 3 October 1995; accepted 19 October 1995

### Methods

### *Synthesis of taxane analogues*

The synthesis of the taxane analogues (Figure 1) has been described in detail previously (Klein *et al.*, 1994; Li *et al.*, 1994). Paclitaxel was obtained from NaPro Biotherapeutics, Boulder, CO, USA.

### In vitro *evaluation of anti-tumour compounds*

The *in vitro* potency of experimental and control taxanes was determined using a colorimetric assay to assess cell cytotoxicity as described previously (Chu *et al.*, 1992). Briefly, tumour cell lines were maintained in RPMI-1640 plus 10% fetal calf serum. Experimental and control taxane compounds were dissolved in ethanol and added to the tumour cells in 96-well microtitre plates. The cells were exposed to compounds for 72 h. Cell viability was determined by MTT dye reduction using absorbency at 470 nm. The inhibitory concentration 50% ($IC_{50}$) was determined as the drug concentration to produce 50% cell cytotoxicty.

### *Animals*

The mice used in the tumour tests were obtained from Harlan Sprague-Dawley, Indianapolis, IN, USA. Female C57BL/6×DBA/2F1 (B6D2F1/Hsd black, hereafter referred to as BDF1) mice were housed ten animals to a cage on bedding and given free access to food and water. Hsd:Athymic Nude-nu (hereafter referred to as nude) mice were housed ten to a

**Figure 1** Structures of the paclitaxel analogues 9-DH-t and 10-DeAc-9-DH-t. A-85576, 9-DH-t; R1 = Ac; A-86415, 10-DeAc-9-DH-t; R1 = H.

cage in sterilised barrier cages and were given free access to sterilised food and water. Outbred CD-1 mice used in pharmacokinetic trials were obtained from Charles Rivers Labs (Wilmington, MA, USA).

*Tumour cells*

P388, B16F10, M109, HT29, A549, and MX-1 tumour cells were obtained from American Type Culture Collection, Rockville, MD, USA.

In vivo *evaluations of anti-tumour compounds*

B16 melanoma solid tumours were harvested from donor mice and homogenised in a 1:20 weight to volume ratio using a tissue homogeniser. (Tekmar, Cincinnati, OH, USA). The resulting brei was injected intraperitoneally (i.p.) into BDF1 mice at 0.5 ml per mouse. This inoculation produced a tumour ascites that was lethal in approximately 20 days in untreated mice. Drug efficacy against i.p. tumour ascites was evaluated by the per cent increase in life span (% ILS) based upon mean survival time (MST) of treated *vs* untreated mice, and on cures evaluated on 60 day survival rates. There were ten mice in each dosage group and 20 untreated control mice in each trial.

For M109 lung tumour trials breis were produced by homogenisation of the solid M109 tumour tissue from the flanks of donor mice. A 1:20 weight to volume brei was prepared in sterile Hanks' balanced salt solution and 0.5 ml was injected subcutaneously (s.c.) into the flanks of BDF1 mice. The solid tumour mass grew to a 1.0 g mass in 10–12 days, and doubled in volume approximately every 2 days in untreated mice. Drug efficacy against M109 subcutaneous tumour inoculations was based upon delay in tumour growth to 1.0 g (delay to 1.0 g), and upon tumour weight inhibition (TWI) of treated tumour mass when control mean tumour mass was approximately 1.0 g. Tumour mass was calculated as $(L \times W^2)/2$. There were ten mice in each dosage group and 20 untreated control mice in each trial.

For xenograft solid tumour models, MX-1 human mammary inoculas were prepared by aseptic homogenisation of solid tumour tissue. A 1:8 weight to volume brei was made with MX-1 tumour tissue and 0.5 ml was injected s.c. into nude mice. The solid tumour mass grew to a 0.5 g mass in 20–30 days and doubled in volume approximately every 7 days in untreated mice. Drug efficacy against s.c. MX-1 xenograft tumour inoculations was based upon delay in tumour growth to 0.5 g (delay to 0.5 g), and TWI in treated mice. The TWI was calculated when mean tumour mass of untreated mice was approximately 0.5 g. There were ten mice in each dosage group and 20 untreated control mice in each trial. Mice were dosed on a Q.D. × 5 schedule based on the MTD data obtained in earlier B16F10 trials.

*Dosing*

The taxanes were dissolved in 100% ethanol, which was then diluted with cremophor to yield a 50:50 mixture. The solutions were diluted with sterile injectable water (Abbott Labs, North Chicago, IL, USA) to yield appropriate concentrations, and were administered in a volume of 0.5 ml i.p. The final volumes of ethanol and cremophor (Sigma, St Louis, MO, USA) combined were no more than 12% (6% each). The initial dose was administered 1 day after tumour inoculation. Against B16F10 and MX-1 tumours the taxane analogues were administered once daily for 5 consecutive days (days 1–5 after inoculation). Against M109 subcutaneous tumours the taxane analogues were administered once daily, every fourth day for a total of three injections (days 1, 5, 9 after inoculation). The schedules for drug administration were adapted from general National Cancer Institute guidelines for preclinical testing of anti-tumour agents (NCI, 1985; Gerari *et al*, 1972). Dosages that caused greater than 20% premature mortality ($\leqslant$75% MST of untreated mice) were classified as toxic. The maximum tolerated dose (MTD) was determined as the highest drug dose administered once daily for 5 consecutive days that produced less than 20% mortality. The trials were performed once.

*Pharmacokinetics*

Pharmacokinetic evaluation was performed on selected compounds in male CD-1 mice (Charles Rivers Labs). The mice were injected i.p. with the taxane compounds at 20 mg $kg^{-1}$. The taxane compounds were formulated as described above, with a final ethanol–cremophor–water concentrations of 6%:6%:88%. At 0.25, 0.5, 1, 2, 4, 8 and 12 h after dosing groups of three mice were exsanguinated by cardiac puncture and blood was collected into heparinised tubes. Plasma was separated from cellular components by centrifugation and was frozen at −70°C until analysis.

The compounds of interest were separated from plasma contaminants by utilising a liquid–liquid extraction with a mixture of ethylacetate and hexane. The samples were evaporated to dryness, reconstituted and then chromatographed on a 5 cm × 4.0 mm 3 μM YMC-C8 column with an acetonitrile–methanol–trifluoroacetic acid (0.1%) in 0.01 M tetramethylammonium perchlorate mobile phase at a flow rate of 1.0 ml $min^{-1}$ with low-wavelength u.v. detection of the analytes at 205 nm. The analytical methods for parent drug were linear (correlation coefficient > 0.99) over the concentration range 0–22 μg $ml^{-1}$ with a mean per cent standard deviation < 3% for the analysis of triplicate mouse plasma standards at six different concentrations. The limit of quantitation from a 0.3 ml plasma sample was estimated to be 0.1 μg $ml^{-1}$ based on recovery of parent drug from spiked samples. Plasma samples with concentrations of drug in excess of the linear range of the standard curve were diluted and reassayed to provide a value within the linear concentration range. The peak plasma concentration ($C_{max}$) and time to peak plasma concentration ($T_{max}$) were derived from the three highest calculated plasma concentrations. The area under the curve (AUC) values were calculated by the trapezoidal method over the time course of the study (0–12 h) using the mean plasma concentration of parent drug at each time point.

*Statistical analysis*

Delay in solid tumour growth for treated and untreated mice was compared using the Student–Neuman–Keuls test following an ANOVA of the tumour growth that rejected the null hypothesis. The chi-square test was used for analysis of per cent cures of treated *vs* untreated mice in the MX-1 trial. A significance level of 0.05 was used for comparisons in the solid tumour trials. In the B16 ascites tumour trial means of survival time were compared using the Student *t*-test for unpaired data with a significance level of 0.05.

### Results

In vitro *cell cytotoxicity*

The paclitaxel analogues A-85576, 9-dihydrotaxol (9-DH-t), and A-86415, 10-deacetyl-9-dihydrotaxol (10-DeAc-9-DH-t), produced $IC_{50}$ values that ranged from equally to 8-fold less potent than paclitaxel (Table I). 10-DeAc-9-DH-t was as

**Table I** *In vitro* tumour cell cytotoxicity of taxane analogues

| Compound | $IC_{50}$ ($ng\,ml^{-1}$)[a] A549 | HT-29 | B16F10 | P388 | Solubility[b] ($\mu g\,ml^{-1}$) |
|---|---|---|---|---|---|
| A-85576 (9-DH-t) | 19.0 | 8.0 | 25 | 53.0 | 226 |
| A-86415 (10-DeAc-9-DH-t) | 11.0 | 1.9 | 39 | 14.0 | 69 |
| Paclitaxel | 3.4 | 2.7 | 4.9 | 9.9 | 2.92 |

[a]ng $ml^{-1}$ to produce 50% cytotoxicity in the indicated tumour cell line. [b]Water solubility potent as paclitaxel against HT29 and P388 tumour cells, but was 3- to 8-fold less potent *vs* A549 and B16F10 cells respectively. 9-DH-t was 3- to 5-fold less potent than paclitaxel against the four tumour cell lines. The water solubility of 9-DH-t was approximately 75-fold greater than paclitaxel. The water solubility of 10-DeAc-9-DH-t was approximately 20-fold that of paclitaxel.

### In vivo *MTD doses in mice*

When administered i.p. once daily for 5 consecutive days, the maximum tolerated dose (MTD) of paclitaxel was 25 mg $kg^{-1}$ $day^{-1}$, while the MTDs of 9-DH-t and 10-DeAc-9-DH-t were greater than 100 mg $kg^{-1}$ $day^{-1}$. The MTDs were determined in BDF mice bearing B16F10 ascites tumours (Table II).

**Table II** *In vivo* lethal dose 50% ($LD_{50}$) and maximum tolerated dose (MTD) values (mg $kg^{-1}$per day) for taxane analogues in mice following single and multiple doses by i.p. route

| *Compound* | *MTD*[a] (*q.d. days 1–5*) |
|---|---|
| A-85576 (9-DH-t) | >100 |
| A-86415 (10-DeAc-9-DH-t) | >100 |
| Paclitaxel | 25 |

[a]Maximum dose (mg $kg^{-1}$per day) that yielded ≤20% lethality following five i.p. doses, administered once daily in BDF mice bearing B16F10 ascites tumours.

### *Efficacy of taxane analogues* vs *i.p. B16F10 murine melanoma*

At non-toxic doses the paclitaxel analogues 9-DH-t and 10-DeAc-9-DH-t optimally produced 39% and 83% increase in life span (ILS) and cure rates of 0% and 10% respectively (Table III). Paclitaxel treatment produced a 96% ILS and a 0% cure rate. Untreated mice survived approximately 20 days. The optimal dosage for paclitaxel was 25 mg $kg^{-1}$ $day^{-1}$ when administered i.p. once daily for 5 consecutive days. On the same schedule the optimal doses of the paclitaxel analogues 9-DH-t and 10-DeAc-9-DH-t were 50 and 100 mg $kg^{-1}$ $day^{-1}$. There was a dose–response effect to paclitaxel and the analogues.

### *Efficacy of taxane analogues* vs *subcutaneous M109 murine solid tumour*

The paclitaxel analogues 9-DH-t and 10-DeAc-9-DH-t yielded 10.5 and 5.1 day delays in solid tumour growth to 1 g (Table IV). Paclitaxel at a non-toxic dose produced only a 0.6 day delay in tumour growth to 1 g. Untreated mice yielded tumours with a mass of 1 g in 12 days. There was a 0% cure rate for all compounds. The tumour weight inhibition. (TWI) when untreated mice had a mean tumour mass of 1 g was 79–90% for the taxane analogues, compared with 47% for mice treated with paclitaxel. When administered i.p on a schedule of every fourth day for three total injections (days 1, 5, 9 after inoculation), the optimal doses of 9-DH-t and 10-DeAc-9-DH-t were 100 mg $kg^{-1}$ injection. There was a general dose reponse to all three compounds.

**Table III** *In vivo* efficacy of 9-dihydrotaxol and 10-deacetyl-9-dihydrotaxol *vs* murine B16F10 melanoma ascites tumour

| *Compound* | *Dose*[a] | *MST*[b] | *%ILS*[c] | *Per cent toxicity*[d] | *Per cent cures*[e] |
|---|---|---|---|---|---|
| A-85576 (9-DT-t) | 100 | 28.7 ± 12.6[f] | 46 | 20 | 0 |
| | 50 | 27.3 ± 4.9[f] | 39 | 0 | 0 |
| | 25 | 23.9 ± 4.3 | 21 | 0 | 0 |
| A-86415 (10-DeAc-9-DH-t) | 100 | 34.8 ± 4.5[f] | 77 | 0 | 0 |
| | 50 | 36.1 ± 4.5[f] | 83 | 0 | 10 |
| | 25 | 30.0 ± 11.0[f] | 52 | 0 | 0 |
| Paclitaxel | 25 | 38.6 ± 4.8[f] | 96 | 0 | 0 |
| | 12.5 | 26.4 ± 3.4[f] | 34 | 0 | 0 |
| | 6.25 | 26.4 ± 3.4[f] | 34 | 0 | |
| Untreated | NA | 19.7 ± 2.7 | NA | NA | NA |

[a]mg $kg^{-1}$per day, q.d., i.p., days 1–5 after inoculation. [b]Mean survival time (days) ± s.d. [c]Group mean increase in lifespan. [d]Percentage of mice with premature death. [e]Percentage of mice alive 60 days after inoculation. [f]Significantly different from untreated mice ($P < 0.05$). NA, not applicable.

**Table IV** *In vivo* efficacy of taxane analogues *vs* murine M109 solid tumour

| *Compound* | *Dose*[a] | *TWI*[b] | *Days (1.0 g)*[c] | *Delay (1.0 g)*[d] | *Per cent toxicity* | *Per cent cures*[e] |
|---|---|---|---|---|---|---|
| A-85576 (9-DH-t) | 100 | 90 | 22.5 ± 7.7[f] | 10.5 | 0 | 0 |
| | 50 | 46 | 14.1 ± 3.2 | 2.1 | 0 | 0 |
| | 25 | 34 | 13.6 ± 2.3 | 1.6 | 0 | 0 |
| A-86415 (10-DeAc-9-DH-t) | 100 | 79 | 17.1 ± 2.6[f] | 5.1 | 0 | 0 |
| | 50 | 19 | 12.0 ± 0 | 0 | 0 | 0 |
| | 25 | 10 | 12.0 ± 0 | 0 | 0 | 0 |
| Paclitaxel | 25 | 47 | 12.9 ± 1.1 | 0.9 | 30 | 0 |
| | 12.5 | 40 | 12.6 ± 1.0 | 0.6 | 0 | 0 |
| | 6.25 | 22 | 12.4 ± 0.8 | 0.4 | 0 | 0 |
| Untreated | NA | NA | 12.0 ± 0 | NA | NA | NA |

[a]mg $kg^{-1}$per day, q.d., i.p., days 1, 5, 9 after inoculation. [b]Per cent tumour weight inhibition when untreated control mean = 1.0 g. [c]Days to reach mean tumour mass = 1.0 g ± s.d. [d]Delay (days) to reach mean tumour mass = 1.0 g, compared with untreated. [e]Percentage of mice with no palpable tumours, day 60. [f]Significantly different from untreated mice ($P < 0.05$). NA, not applicable.

*Efficacy of taxane analogues* vs *human MX-1 mammary solid tumour xenograft*

The taxane analogues 9-DH-t and 10-DeAc-9-DH-t produced 21 and 33 day delays in tumour growth compared with untreated controls (Table V). When administered once daily on days 1–5 after inoculation there was a 60% and 70% cure rate for 9-DH-t and 10-DeAc-9-DH-t. The optimal dose for these two compounds was 100 mg $kg^{-1}$ per dose on this schedule. Paclitaxel yielded a 13.4 day delay in MX-1 tumour growth and a 10% cure rate compared with untreated animals. The optimal dosage for paclitaxel was 12.5 mg $kg^{-1}$ per dose when administered on days 1–5 after inoculation *vs* MX-1 tumour in nude mice.

The two analogues optimally produced a tumour weight inhibition of 98–100% against MX-1 when untreated mice had a mean tumour mass of 0.5 g. Paclitaxel yielded a TWI value of 80% at non-toxic doses.

*Pharmacokinetic properties of taxane analogues following i.p. dosing in mice*

9-DH-t yielded $C_{max}$ values of 40 $\mu g\ ml^{-1}$ plasma at 0.5 h after dosing (Table VI). The AUC value of over 75 $\mu g\ h^{-1}\ ml^{-1}$ for 9-DH-t was the largest value observed for the three compounds. 10-DeAc-9-DH-t produced a $C_{max}$ of 11 $\mu g\ ml^{-1}$ at 1.5 h after administration. The AUC value for 10-DeAc-9-DH-t was 30 $\mu g\ h^{-1}\ ml^{-1}$. Paclitaxel yielded a $C_{max}$ of 14 $\mu g\ ml^{-1}$ at 1.5 h after administration and an AUC value of 43 $\mu g\ h^{-1}\ ml^{-1}$. The $t_{1/2}$ values for 9-DH-t, 10-DeAc-9-DH-t and paclitaxel were 1.9, 2.8, 2.9 h.

### Discussion

The two taxane analogues showed favourable solubility, toxicity and efficacy profiles relative to paclitaxel. Both 10-DeAc-9-DH-t and 9-DH-t demonstrated less toxicity than paclitaxel, and considerable efficacy *vs* murine and human solid tumours *in vivo*. A decrease in lethal toxicity allowed higher daily doses to be administered for the analogues. The *in vitro* potency of the taxane analogues was within one log of that of paclitaxel, but there was no clear correlation between *in vitro* potency and *in vivo* efficacy.

The mouse models of tumour growth are valid indicators of potential clinical utility of paclitaxel analogues. Both ascites and solid tumour mouse models are commonly used as screens for preclinical efficacy (Gerari *et al.*, 1972). Efficacy *vs* solid tumours has gained prominence as a means of testing for an unmet clinical need, compared with efficacy *vs* ascites tumours. For paclitaxel analogues efficacy in human solid tumour xenograft models is considered an indicator for preclinical efficacy. Paclitaxel efficacy *vs* murine tumour lines tended to be less than that *vs* human tumours. While effective therapy against murine or human xenografted tumours does not ensure clinical success, efficacy in these models gives credibility to the clinical potential of taxane analogues.

Both of the analogues demonstrated efficacy that compared favourably with paclitaxel *vs* murine M109 and human MX-1 solid tumour xenograft. The efficacy of paclitaxel *vs* M109 in these trials was less than that reported previously (Rose, 1981, 1991). However, different routes of administration and formulations of paclitaxel were used in the other studies. These studies tested the taxane analogues in equal formats for purposes of an initial *in vivo* comparison. Efficacy *vs* solid tumours is an important preclinical marker for potential clinical efficacy of anti-tumour drugs.

The efficacy of the taxane analogues *vs* ascites B16 tumour provided additional evidence for activity of the analogues, since paclitaxel is more reliably effective against i.p. tumours than against s.c. tumours (Rose, 1981; Lavelle *et al.*, 1989). There was no clear correlation between *in vitro* potency and *in vivo* efficacy *vs* B16 ascites tumour. Against B16 ascites 10-DeAc-9-DH-t was equal to or better than paclitaxel, while 9-DH-t was less effective.

The i.p. pharmacokinetic studies in mice were used as a basic test of bioavailability in the tumour test system, rather than as an exhaustive pharmacokinetic analysis. The efficacy of paclitaxel therapy is dependent upon the route of administration; with i.p. dosing paclitaxel therapy is usually effective against ascites tumours (Rose, 1981). The high $C_{max}$ and AUC values attained following i.p. dosing suggest good

**Table V** *In vivo* efficacy of taxane analogues *vs* human MX-1 xenograft solid tumour

| *Compound* | *Dose*[a] | *TWI*[b] | *Days to 1.0 g*[c] | *Delay (1.0 g)*[d] | *Per cent toxicity* | *Per cent cures*[e] |
|---|---|---|---|---|---|---|
| A-85576 (9-DH-t) | 100 | 98 | 49.0±17.0[f] | 21.1 | 0 | 60[f] |
| | 50 | 94 | 40.3±3.5[f] | 12.4 | 0 | 50[f] |
| | 25 | 65 | 33.8±5.5 | 5.9 | 0 | 0 |
| | 12.5 | 31 | 29.5±7.6 | 1.6 | 0 | 0 |
| A-86415 (10-DeAc-9-DH-t) | 100 | 100 | 61.0±0[f] | 33.1 | 0 | 70[f] |
| | 50 | 99 | 42.6±7.7[f] | 14.7 | 0 | 30 |
| | 25 | 73 | 33.3±7.6 | 5.4 | 0 | 10 |
| | 12.5 | 27 | 28.0±5.7 | 0 | 0 | 20 |
| Paclitaxel | 25 | 100 | 47.0±0[f] | 19.1 | 40 | 30 |
| | 12.5 | 80 | 41.3±9.7[f] | 13.4 | 0 | 10 |
| | 6.25 | 42 | 29.3±5.0 | 1.4 | 0 | 0 |
| Untreated | NA | NA | 27.9±5.0 | NA | NA | NA |

[a]mg $kg^{-1}$ per day, q.d., i.p., days 1–5 after inoculation. [b]Percent tumour weight inhibition when untreated control mean = 0.5 g. [c]Days to reach mean tumour mass = 0.5 g ± s.d. [d]Delay (days to reach mean tumour mass = 0.5 g, compared with untreated. [e]Percentage of mice with no palpable tumours, day 60. [f]Significantly different from untreated mice ($P<0.05$). NA, not applicable.

**Table VI** Pharmacokinetic properties of 9-dihydrotaxane analogues in mice

| *Compound*[a] | $C_{max}$ ($\mu g\ ml^{-1}$) | Tmax (h) | $t_{\frac{1}{2}}$ (h) | *AUC* ($\mu g$ *per h* $ml^{-1}$) |
|---|---|---|---|---|
| A-85576 (9-DH-t) | 40.32 | 0.58 | 1.9 | 75.62 |
| A-86415 (10-DeAc-9-DH-t) | 11.19 | 1.40 | 2.8 | 29.81 |
| Paclitaxel | 14.13 | 1.50 | 2.9 | 42.53 |

[a]Compounds dosed at 20 mg $kg^{-1}$ i.p.

bioavailability for both analogues. The serum levels and AUC values of 9-DH-t were greater than paclitaxel, while the serum levels of 10-DeAc-9-DH-t was less than paclitaxel at equal dosages. The $t_{1/2}$ values for 9-DH-t and paclitaxel were similar (2.8 and 2.9 h), suggesting a potential dosing advantage for the analogue based on the decrease in toxicity. Pharmacokinetic studies in mice must be interpreted with caution owing to the higher variability between animals.

The decrease in apparent toxicity of the analgoues relative to paclitaxel was encouraging. The analogues were tolerated at daily i.p. doses of 100 mg $kg^{-1}$ $day^{-1}$, which was 4-fold greater than paclitaxel. This decrease in toxicity allowed the analogues to be administered at higher doses, resulting in greater efficacy. Some reports suggest that daily doses of paclitaxel may be superior to larger, less frequent doses (NCI, 1985). Both 9-DH-t and 10-DeAc-9-DH-t were better tolerated than paclitaxel. In general, nude mice did not tolerate daily doses of the taxane analogues as well as other mice and paclitaxel showed variability in maximum tolerated dose between trials. In the M109 and MX-1 solid tumour trials paclitaxel at 25 mg $kg^{-1}$ was not tolerated (30% and 40% mortality), while daily doses of 25 mg $kg^{-1}$ were tolerated in the B16 ascites tumour trials. The optimal balance of efficacy and toxicity has not been yet been determined with taxane analogues.

The efficacy of the taxane analogues relative to paclitaxel suggests potential clinical utility. Paclitaxel has demonstrated clinical efficacy *vs* human breast and ovarian tumours (McGuire *et al.*, 1989; Thigpen *et al.*, 1990). Reponse rates of 48% were recorded in breast tumour patients who had failed one previous course of chemotherapy (Holmes *et al.*, 1991). Paclitaxel has been used against other tumours, including leukaemia and malignant melanoma (Einzig, 1988; Rowinsky *et al.*, 1989). A 30% response rate against ovarian tumours that had failed cisplatinum therapy was obtained with paclitaxel (McGuire *et al.*, 1989). The clinical use and efficacy of docetaxel has generated additional support for this base structure (Chabner *et al.*, 1991; Ringel and Horwitz, 1993). The preclinical efficacy and toxicity profiles of the taxane analogues in relation to paclitaxel may be extended to project potential clinical use.

In summary, two taxane analogues were tested for efficacy in mouse models of solid and ascites tumours. These analogues were generally superior or similar to paclitaxel in efficacy. The analogues 9-DH-t and 10-DeAc-9-DH-t yielded less toxicity and greater water solubility than paclitaxel. The preclinical efficacy of these two compounds supports further development.

### Abbreviations

9-DH-t, 9-dihydrotaxol; 10-DeAc-9-DH-t, 10-deacetyl-9-dihydrotaxol; $IC_{50}$, 50% inhibitory concentration; %ILS, per cent increase in lifespan; TWI, tumour weight inhibition; $C_{max}$, maximum serum concentration; $T_{max}$, time to maximum concentration; AUC, area under the curve; $LD_{50}$, 50% lethal dose; MTD, maximum tolerated dose.

### Acknowledgements

The work of Darlene Balli (cell cytotoxicity), Mike Mitten, Andy Oleksijew, Lenette Paige and Tom Hutch (*in vivo*), Kevin Garren (solubility), and Alan Dutkiewicz, Mike Nukkula and Donna Strasburg (Animal Care) is gratefully acknowledged.

### References

BISSERY M-C, GUENARD D, GUERITTE-VOEGELEIN F AND LAVELLE F. (1991). Experimental antitumor activity of taxotere (RP 56976, NCS 628503), a paclitaxel analogue. *Cancer Res.*, **51**, 4845–4852.

CHABNER BA. (1991). Paclitaxel. In *Principles and Practice of Oncology*, Devitta VT, Hellman S and Rosenbert SA (eds) pp. 1–10. JB Lippincott: Philadelphia.

CHU D T W, HALLAS R, CLEMENT J J, ALDER J, MCDONALD E AND PLATTNER JJ. (1992). Synthesis and antitumor activities of quinolones antiplastic agents. *Drug Exp. Clin. Res.*, **18**, 275–282.

EINZIG AL, TRUMP DL, SASLOFF J, GOROWSKI E, DUTCHER J AND WIERNIK PH. (1988). Phase II pilot study of paclitaxel in patients with malignant melanoma. *Proc. Am. Soc. Clin. Onco.*, **7**, 249–253.

FUCHS D A AND JOHNSON RK. (1978). Cytologic evidence that paclitaxel, an antineoplastic agent from *Taxus brevifolia*, acts as a mitotic spindle poison. *Cancer Treat. Rep.*, **62**, 1219–1222.

GERAN RI, GREENBERG RH, MACDONALD MM, SCHUMACHER AM AND ABBOTT BJ. (1972). Protocols for screening chemical agents and natural products against animal tumours and other biologic systems. *Cancer Chemother. Rep.*, **3**, 1–103.

GUNAWARDANA GP, PREMACHANDRAN U, BURRES NS, WHITTEN DN, HENRY R, SPATON S AND MCALPINE JB. (1992). Isolation of 9-dihydro-13-acetylbaccatin III from *Taxus canadensis J. Natl. Prod.*, **55**, 1686–1689.

HOLMES FA, FRYE D, THERIAULT RL, WALTERS RS, FOREMAN AD, NEWTON LK, BUZDAR AU AND HORTOBAGYI GN. (1991). Phase II study of paclitaxel in patients with metastatic breast cancer. *Proc. Am. Soc. Clin. Oncol.*, **10**, 60.

KLEIN LL. (1993). Synthesis of 9-dihydrotaxol: a novel bioactive taxane. *Tetrahedron Lett.*, **34**, 2047–2050.

KLEIN LL, MARING C, LI L, YEUNG CM, THOMAS SA, GRAMPOVNIK DJ, PLATTNER JJ AND HENRY RF. (1994). Synthesis of ring B-rearranged taxane analogues. *J. Org. Chem.*, **59**, 2370–2373.

LAVELLE F, FIZAMES C, GUERITTE-VOEGELEIN F, GUENARD D AND POTIER P. (1989). Experimental properties of RP 56976, a paclitaxel derivative. *Proc. Am. Assoc. Cancer Res.*, **30**, 566.

LI L, THOMAS SA, KLEIN LL, YEUNG CM, MARING C, GRAMPOVNIK DJ, LARTEY P AND PLATTNER JJ. (1994). Synthesis and biologic evaluation of C3′ modified analogues of 9 (R) dihydrotaxol. *J. Medic. Chem.*, **37**, 2655–2663.

MCGUIRE WP, ROWINSKY EK, ROSENSHEIN NB, GRUMBINE FC, ETTINGER DS, ARMSTRONG DK AND DONEHOWER DC. (1989). Paclitaxel: a unique antineoplastic agent with significant activity in advanced ovarian epithelian neoplasms. *Ann. Intern. Med.*, **111**, 273–279.

NATIONAL CANCER INSTITUTE. (1985). *Developmental Therapeutics Program Instruction*, no. 14. Division of Cancer Treatment, Information Technology Branch, NCI: Bethesda, MD.

NATIONAL CANCER INSTITUTE. (1990). *Paclitaxel (IND 22850, NSC 12973)*. Clinical brochure. Division of Cancer Treatment, NCI: Bethesda, MD.

RINGEL I AND HORWITZ SB. (1991). Studies with RP 56976 (Taxotere): a semisynthetic analogue of paclitaxel. *J. Natl Cancer Inst.*, **83**, 288–291.

ROSE WC. (1981). Evaluation of Madison 109 lung carcinoma as a model for screening antitumor drugs. *Cancer Treat.*, **65**, 299–312.

ROSE WC. (1991). Paclitaxel: a review of its preclinical *in vivo* antitumor activity. *Anti-Cancer Drugs*, **3**, 311–321.

ROWINSKY EK, BURKE PJ, KARP JE, TUCKER RW, ETTINGER DS AND DONEHOWER RC. (1989). Phase I and pharmacodynamic study of paclitaxel in refractory acute leukemias. *Cancer Res.*, **49**, 4640–4647.

SCHIFF PB, FANT J AND HORWITZ SB. (1979). Promotion of microtubule assembly *in vitro* by paclitaxel. *Nature*, **22**, 665–667.

SCHIFF SB. (1980). Paclitaxel stabilizes microtubules in mouse fibroblast cells. *Proc. Natl. Acad. Sci. USA*, **77**, 1561–1565.

THIGPEN T, BLESSING J AND BALL H. (1990). Phase II trial of paclitaxel as a second line therapy for ovarian carcinomas: A gynecologic oncology group study. *Proc. Am. Soc. Clin. Oncol.*, **9**, 604.

WANI MC, TAYLOR HL, WALL ME, COGGON P AND MCPHAIL AT. (1971). Plant antitumor agents. VI. The isolation and structure of paclitaxel, a novel antileukemic and antitumor agent from Taxus brevifolia. *J. Am. Chem. Soc.*, **93**, 2325–2327.